# Analysis of Risk Factors for Non-union After Surgery for Limb Fractures: A Case-Control Study of 669 Subjects

**DOI:** 10.3389/fsurg.2021.754150

**Published:** 2021-12-14

**Authors:** Kun Quan, Qiang Xu, Meisong Zhu, Xuqiang Liu, Min Dai

**Affiliations:** Department of Orthopedics, The First Affiliated Hospital of Nanchang University, Artificial Joints Engineering and Technology Research Center of Jiangxi Province, Nanchang, China

**Keywords:** limb fractures, bone non-union, fracture healing, risk factors, multivariate logistic regression

## Abstract

**Objective:** The purpose of this study was to analyze the risk factors for limb fracture non-union in order to improve non-union prevention and early detection.

**Methods:** A total of 223 patients with non-union after surgery for limb fractures performed at our institution from January 2005 to June 2017 were included as the case group, while a computer-generated random list was created to select 446 patients with successful bone healing after surgery for limb fractures who were treated during the same period as the control group, thus achieving a ratio of 1:2. The medical records of these patients were reviewed retrospectively. Age, sex, body mass index, obesity, smoking, alcohol, diabetes, hypertension, osteoporosis, fracture type, multiple fractures, non-steroidal anti-inflammatory drugs (NSAIDs) use, delayed weight bearing, internal fixation failure, and infection data were analyzed and compared between the two groups. A multivariate logistic regression model was constructed to determine relevant factors associated with non-union.

**Results:** After comparison between two groups by univariate analysis and multivariate logistic regression, we found some risk factors associated that osteoporosis (odds ratio [OR] = 3.16, 95% confidence interval [CI]: 2.05–4.89, *p* < 0.001), open fracture (OR = 2.71, 95%CI: 1.72–4.27, *p* < 0.001), NSAIDs use (OR = 2.04, 95%CI: 1.24–3.37, *p* = 0.005), delayed weight bearing (OR = 1.72, 95%CI: 1.08–2.74, *p* = 0.023), failed internal fixation (OR = 5.93, 95%CI: 2.85–12.36, *p* < 0.001), and infection (OR = 6.77, 95%CI: 2.92–15.69, *p* < 0.001) were independent risk factors for non-union after surgery for limb fractures.

**Conclusions:** Osteoporosis, open fracture type, NSAIDs use, delayed weight bearing, failed internal fixation, and infection were found to be the main causes of bone non-union; clinicians should, therefore, take targeted measures to intervene in high-risk groups early.

## Introduction

Recently, the number of patients with successful callus formation after fracture surgical management has decreased, with 5–10% of non-union cases leading to worrying outcomes (1, 2). The loss of productivity caused by a long healing process affects patients, healthcare systems, and the economy (3). It is estimated that 100,000 fracture cases show non-union in the United States each year, with the cost of non-union complications being close to $12,000 per patient (4, 5). In addition, in the United Kingdom, the cost of treating non-union is estimated at £7,000 to £79,000, but these figures relate only to the cost of hospitalization (6, 7), which indicates that a large amount of medical resources are consumed each year in non-union cases.

Risk factors can determine which patients may benefit from more active interventions among those who tend to develop non-union, and a clear idea of the risk of non-union may help to choose between competing treatment options (8). Identifying these factors will enable surgeons to identify high-risk cases before surgery in order to implement measures to promote healing, such as more comprehensive metabolic examinations, medical interventions, early bone grafting, and the use of osteoinductive agents and bone stimulants (9). Furthermore, these findings will help surgeons consult on the anticipated outcomes of such patients. Bone non-union is a multifactorial disease, and it is considered that the coexistence of different risk factors is crucial to its occurrence. A series of factors have been considered to be the causes of non-union, which can be divided into two main categories: (1) patient-related factors (8), including age and sex, body mass index (BMI), obesity, smoking, alcohol, and medical history of diabetes, hypertension, and osteoporosis; and (2) injury characteristics and treatment-related factors (10), including fracture type, use of non-steroidal anti-inflammatory drugs (NSAIDs), delayed weight bearing, mechanical instability resulting in internal fixation failure, and infection.

Until now, although a large number of studies have been conducted to analyze the multiple risk factors involved in the development of fracture non-union, they are limited by small sample sizes and incomplete potential risk factors (11–14). Moreover, clinical trials have shown that changes in the rate of non-union are related to different surgical treatments (15, 16). However, the above factors cannot fully explain the occurrence of non-union. Most of these studies reported fractures at specific sites, such as the humerus, femur, or tibia, or were limited to one type of non-union, such as that caused only by biological or treatment-related factors, without a systematic and global analysis that is required for limb fractures.

The purpose of this study was, therefore, to investigate the association between the occurrence of non-union after surgery for limb fractures and patient-, injury-, and treatment-related factors. A thorough understanding of these factors and their relative impact may provide better insight into the causes of non-union and the required treatment. Modifiable factors, such as use of NSAIDs and delayed weight bearing, should be strictly evaluated to optimize the patient prognosis.

## Materials and Methods

### Research Data

We retrospectively analyzed 223 patients with bone non-union after surgery for limb fractures in our institution from January 2005 to June 2017 (case group), and created a computer-generated random list to select 446 patients with successful bone healing after surgery for limb fractures (control group), thus achieving a case-control ratio of 1:2. Both groups of patients were treated during the same period. The present study was approved by the Ethics Committee of the First Affiliated Hospital of Nanchang University.

### Inclusion and Exclusion Criteria

The inclusion criteria were as follows: (i) meeting the diagnostic criteria for bone non-union for the case group: fracture not completely healed within 9 months after injury and showing no progression toward healing on radiographs for 3 consecutive months; (ii) a clear history of trauma and (iii) fracture of the humerus, ulna, radius, femur, tibia, or fibula. The exclusion criteria were as follows: (i) comorbid severe loss of other major organs, severe malnutrition, or malignant disease; (ii) severe bone defect requiring bone grafting and (iii) incomplete clinical data.

### Criteria of Related Risk Factors and Data Collection

All cases of limb fractures were treated surgically by experienced doctors in our institution. Postsurgically, the use of NSAIDs such as flurbiprofen and celecoxib was decided according to the patients' pain. Delayed weight bearing was defined as starting weight bearing more than 12 weeks after surgery, and failure of internal fixation was defined as screw loosening, screw fracture, plate fracture, intramedullary nail fracture, and internal fixation rejection. Additionally, all patients were followed up at least 9 months after surgery, which included clinical data and X-ray examinations to observe wound healing, internal fixation, fracture healing, and joint function. The collected patient-related data included age, sex, BMI, obesity, smoking, alcohol, diabetes, hypertension, osteoporosis, fracture type, multiple fractures, use of NSAIDs, delayed weight bearing, failed internal fixation, and infection to further analyze risk factors for non-union.

### Statistical Analyses

Continuous variable data are presented as mean ± standard deviation, and discrete variable data are presented as frequencies. The Shapiro-Wilk test was used to test the normality of continuous variables, and the independent-samples *t*-test was used to compare differences in age and BMI between the case and the control groups. The chi-square test and Spearman correlation coefficient were used to test and analyze differences in other data. The most significant risk factors for bone non-union were analyzed by univariate analysis, and then the risk factors with *P* < 0.05 were analyzed by Logistic regression analysis. All the above analyses were carried out using SPSS 26.0 statistical software (IBM Corp., Armonk, NY, USA). *P* < 0.05 were considered statistically significant.

## Results

### General Characteristics

As shown in Table 1, there were 223 patients in the case group with an average age of 39.8 ± 14.7 years (167 males and 56 females) and 446 patients in the control group with an average age of 38.5 ± 14.9 years (313 males and 133 females). There was no significant difference in average age and sex ratio between the two groups. The average BMI of patients in the case group was higher than that of patients in the control group (23.0 ± 2.6 kg/m^2^ vs. 23.1 ± 2.3 kg/m^2^), but this difference was not significant (*P* = 0.732). There were also no significant differences in smoking, alcohol, diabetes, and hypertension between the two groups (Table 1). Conversely, the proportion of patients with a history of osteoporosis was significantly higher in the case group than in the control group [35.4% (79/223) vs. 12.8% (57/446); *P* < 0.001].

**Table 1:** Characteristics of the patient-related factors.

**Patient characteristics**	**Case group**	**Control group**	***p*** **-value**
	**(***n*** = 223)**	**(***n*** = 446)**	
Age, mean years (±SD)	39.8 ± 14.7	38.5 ± 14.9	0.274
BMI, kg/m^2^	23.0 ± 2.6	23.1 ± 2.3	0.732
Sex			0.202
Male	167 (74.9%)	313 (70.2%)	
Female	56 (25.1%)	133 (29.8%)	
Smoking (*n*, %)			0.359
Yes	31 (13.9%)	51 (11.4%)	
No	192 (86.1%)	395 (88.6%)	
Alcohol (*n*, %)			0.326
Yes	18 (8.1%)	27 (6.1%)	
No	205 (91.9%)	419 (93.9%)	
Medical history (*n*, %)			
Diabetes			0.659
Yes	7 (3.1%)	17 (3.8%)	
No	216 (96.9%)	429 (96.2%)	
Hypertension			0.735
Yes	13 (5.8%)	29 (6.5%)	
No	210 (94.2%)	417 (93.5%)	
Osteoporosis			<0.001
Yes	79 (35.4%)	57 (12.8%)	
No	144 (64.6%)	389 (87.2%)	

### Follow-Up Results

**Figure 1:**
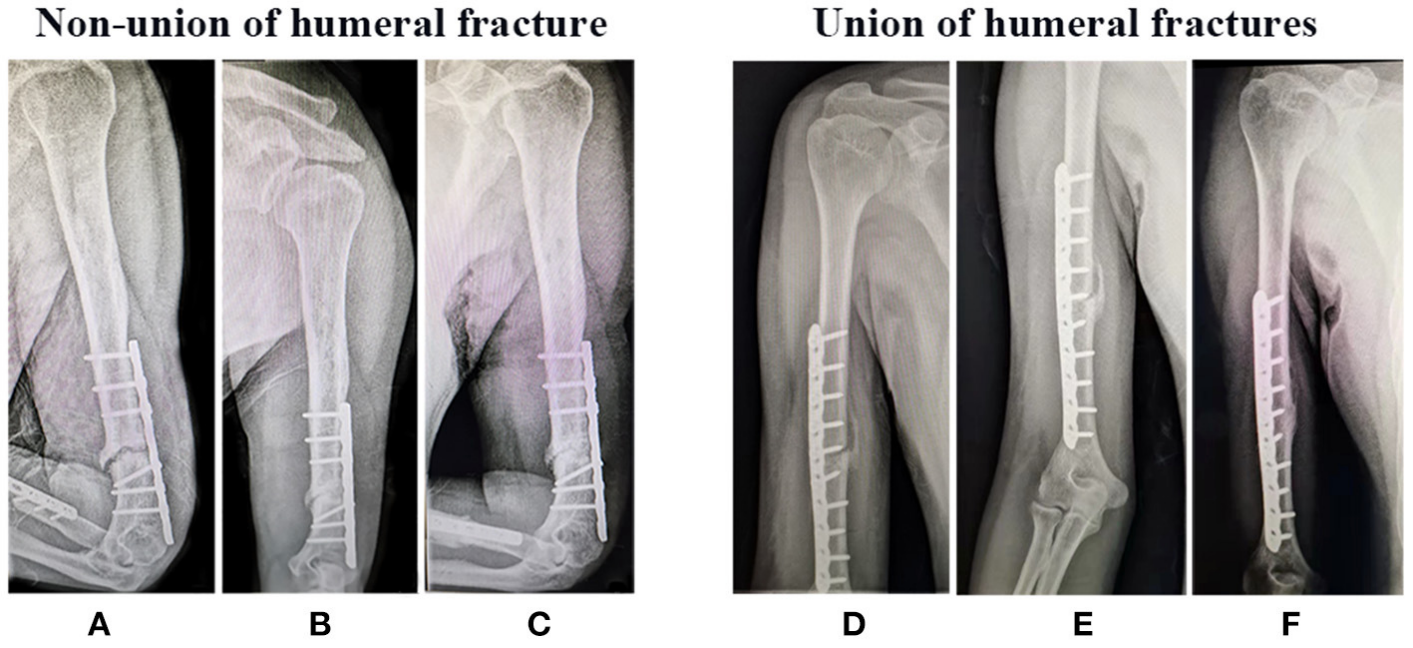
Non-union and Union of humeral fractures after surgery, respectively. X-ray examination **(A,D)** 1 month, **(B,E)** 3 month, and **(C,F)** 9 month after surgery.

**Figure 2:**
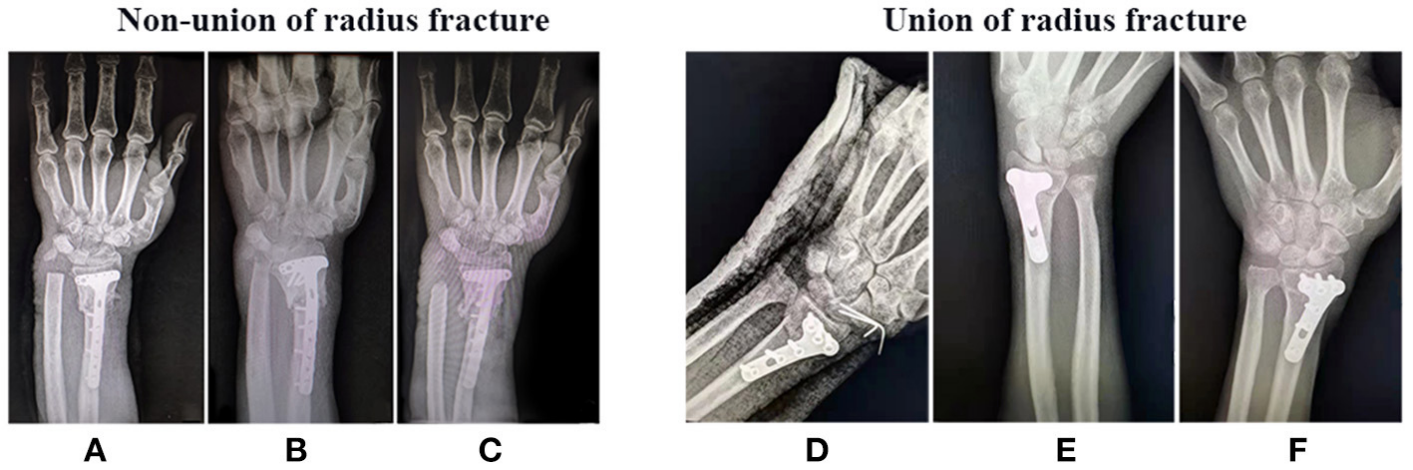
Non-union and Union of radius fracture after surgery. X-ray examination **(A,D)** 1 month, **(B,E)** 3 month, and **(C,F)** 9 month after surgery.

**Figure 3:**
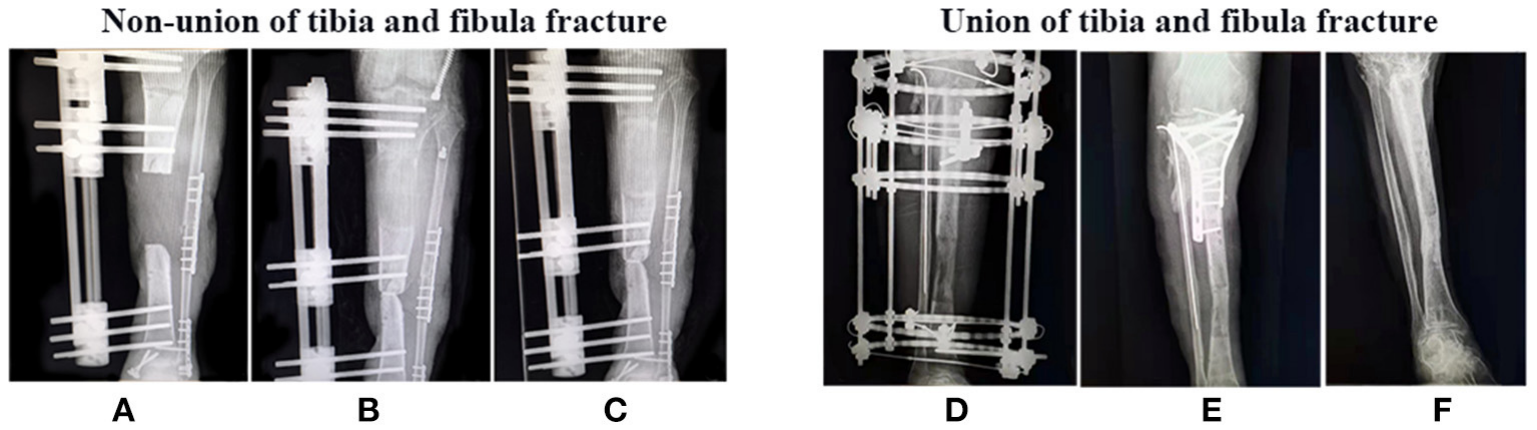
Non-union and Union of tibia and fibula fracture after surgery. X-ray examination **(A,D)** 1 month, **(B,E)** 3 month, and **(C,F)** 9 month after surgery.

All patients were followed up for at least 9 months after surgery, including clinical data, especially x-ray examinations to observe whether the fracture has healed. As shown in Figures 1–3, the typical imaging follow-up data of three typical non-union and union after surgery for limb fractures.

### Outcome of Univariate Analysis

A univariate comparison between the case group and the control group found that fracture type (*P* < 0.001), NSAIDs use (*P* < 0.001), delayed weight bearing (*P* < 0.001), failed internal fixation (*P* < 0.001), and infection (*P* < 0.001) demonstrated a statistically significant difference (Table 2). There was no statistically significant difference only in multiple fracture (*P* = 0.824, Table 2).

**Table 2:** Injury characteristics and treatment-related factors.

**Variable**	**Case group**	**Control group**	***p*** **-value**
	**(***n*** = 223)**	**(***n*** = 446)**	
Fracture type			<0.001
Open	76 (34.1%)	54 (12.1%)	
Closed	147 (65.9%)	392 (87.9%)	
Multiple fracture			
Yes	15 (6.7%)	28 (6.3%)	0.824
No	208 (93.3%)	418 (93.7%)	
NSAID			<0.001
Yes	66 (29.6%)	44 (9.9%)	
No	157 (70.4%)	402 (90.1%)	
Delayed weight bearing			<0.001
Yes	69 (30.9%)	66 (14.8%)	
No	154 (69.1%)	380 (85.2%)	
Failed internal fixation			<0.001
Yes	44 (19.7%)	12 (2.7%)	
No	179 (80.3%)	434 (97.3%)	
Infection			<0.001
Yes	36 (16.1%)	8 (1.8%)	
No	187 (83.9%)	438 (98.2%)	

### Outcome of Multivariate Logistic Regression Analysis

Furthermore, the variables *P* < 0.05 in univariate analysis were selected for further Logistic regression analysis. The results of multivariate analysis indicated that osteoporosis (odds ratio [OR] = 3.16, 95% confidence interval [CI]: 2.05–4.89, *p* < 0.001), open fracture (OR = 2.71, 95%CI: 1.72–4.27, *p* < 0.001), NSAIDs use (OR =2.04, 95%CI:1.24–3.37, *p* = 0.005), delayed weight bearing (OR = 1.72, 95%CI: 1.08–2.74, *p* = 0.023), failed internal fixation (OR = 5.93, 95%CI: 2.85–12.36, *p* < 0.001), and infection (OR = 6.77, 95%CI: 2.92–15.69, *p* < 0.001) were independent risk factors for non-union after surgery for limb fractures (Table 3).

**Table 3:** Multivariable logistic regression model for predictors of non-union.

**Variable**	**Odds ratio estimates (95% Cl)**	***p*** **-value**
Osteoporosis	3.16 (2.05–4.89)	<0.001
Open fracture	2.71 (1.72–4.27)	<0.001
NSAID	2.04 (1.24–3.37)	0.005
Delayed weight bearing	1.72 (1.08–2.74)	0.023
Failed internal fixation	5.93 (2.85–12.36)	<0.001
Infection	6.77 (2.92–15.69)	<0.001

## Discussion

In this study, we found six predictors of non-union after surgery for limb fractures: osteoporosis, open fracture, NSAIDs use, delayed weight bearing, failed internal fixation, and infection. Other patient- and injury-related factors, such as age, sex, BMI, obesity, smoking, alcohol, hypertension, diabetes, closed fracture, and multiple fractures, were not significant risk factors.

### Risk Factors

Without controlling for potential confounding factors, it is not clear whether age and sex itself are risk factors for bone non-union. Although the number of patients included in this study is relatively large, it is not sufficient to control the confounding effect of age and sex. Some studies have reported that age and sex are positively correlated with the risk of non-union (17, 18), while other reports show that there is no significant correlation (19, 20). It is worth noting that the average age of the population in this study is only ~40 years old, and the results may not apply to older patients. This may also explain why age-related factors such as obesity, smoking, alcohol, hypertension, and diabetes have not been identified as statistically significant risk factors in this study, despite being suggested as possible causes of bone non-union in previous studies (21–25). In addition, the study by Solomon et al. showed that there was a significant positive correlation between osteoporosis and the risk of non-union (26). However, another case-control study evaluated 1,498 patients and failed to find such an association (27). Our results indicated that the osteoporosis (OR = 3.16, 95%CI: 2.05–4.89) is associated with non-union, which is in support of the findings of Solomon et al.

In our study, no significant correlation was found between the presence of multiple fractures and non-union. On the contrary, a previous study showed that non-union seems to be associated with multiple fractures (28). This discrepancy may be related to the small proportion of non-union (*n* = 15, 6.7%) in patients with multiple fractures in our study; further studies with larger samples are needed to provide a more definitive understanding of the role of multiple fractures. However, a significant proportion of non-union cases occurred in patients with open fractures (*n* = 76, 34.1%), and the multiple regression analysis showed that patients with open fractures have a greater risk of non-union, which was consistent with the results of a previous study (29). Compared with closed fractures, open fractures are associated with relatively greater trauma, more serious destruction of soft tissue blood supply, higher probability of infection, and decreased blood supply, thus affecting bone healing (30). This also explains that patients with open fractures have a relatively high rate of healing disturbances.

Basic studies have shown that NSAIDs use affects bone healing, mainly due to their inhibiting effect on cyclooxygenase-2, which delays fracture healing (31). A recent retrospective clinical study of 1,900 patients with long bone fractures showed that the postoperative use of NSAIDs doubled the risk of healing complications (32). Moreover, Giannoudis et al. have demonstrated that there is a highly significant relationship between NSAIDs use and bone non-union (*P* < 0.001) (33). Our findings suggest that the NSAIDs use after surgery for limb fractures is associated with a 2.04-fold higher odds ratio of non-union, which is in line with the results of previous studies.

It is crucial to start weight bearing after a fracture since it helps maintain bone and muscle mass and helps restore the performance of activities of daily living. Additionally, weight bearing promotes bone healing through a process called mechanical transduction (34). Recently, a study demonstrated the safety of early weight bearing after fracture surgery (35). We identified a significant association (*P* < 0.001) between delayed weight bearing after surgery and the development of non-union, which is consistent with the results of the study by Westgeest et al. (36). Furthermore, delayed weight bearing was identified as an independent risk factor for the development of non-union in the multivariate analysis. This finding may be explained by a relationship between weight bearing and healing outcomes in the reverse direction. That is, bone non-union may cause more pain, resulting in delayed weight bearing, rather than delayed weight bearing causing non-union (37). Additionally, internal fixation failure is often considered as a reason to postpone the initial weight bearing. In this study, internal fixation failure (*n* = 44, 19.7%) accounted for a large proportion of bone non-union cases, which indicates that there is some association between failed internal fixation and delayed weight bearing. Indeed, our results showed that failed internal fixation (OR = 5.93, 95% CI: 2.85 to 12.36) significantly increases the risk of non-union.

Different opinions have been put forward on whether there is a connection between infection and non-union. Most scholars believe that infection does not increase the risk of non-union (38–40). However, a prospective cohort study evaluated 736 patients and showed that infection was significantly associated with non-union (36). The results of our analysis show that infection is an independent risk factor for non-union, with a 6.77-fold higher risk of non-union in patients with infection. There was also a significant correlation between infection and non-union. This may be due to the increased fracture end necrosis and vascular embolism, giving rise to poor local bone blood supply, so that the formation of bone is disturbed and healing is impaired, ultimately resulting in bone non-union (9).

### Limitations of the Study

Despite these findings, this study has some limitations. First, although we designed a 1:2 matched case-control study to try to minimize the impact of the small sample size, our sample may not fully represent all postoperative patients with limb fractures and may also not be large enough to make our regression analysis conclusive. Second, multiple surgeons were involved in this retrospective study, each with a slightly different non-union definition and treatment. Finally, most patients in this study were middle-aged; thus, it is difficult to apply these findings to older patients. Therefore, further research in a wider age range is needed.

## Conclusions

In summary, this study demonstrated that osteoporosis, open fractures, delayed weight bearing, NSAIDs use, failure of internal fixation, and infection are independent risk factors for non-union. Clinically, surgeons should understand these risk profiles in order to effectively guide patients and successfully set appropriate expectations.

## Data Availability Statement

The original contributions presented in the study are included in the article/supplementary material, further inquiries can be directed to the corresponding author/s.

## Ethics Statement

The studies involving human participants were reviewed and approved by the First Affiliated Hospital of Nanchang University. The patients/participants provided their written informed consent to participate in this study.

## Author Contributions

KQ: methodology, investigation, and writing-original draft. QX: investigation and writing—original draft. MZ: formal analysis. XL: supervision, writing—review and editing, and conceptualization. MD: writing—review and editing, conceptualization, and funding acquisition. All authors contributed to the article and approved the submitted version.

## Funding

This work was supported by the National Natural Science Foundation (No. 81860404).

## Conflict of Interest

The authors declare that the research was conducted in the absence of any commercial or financial relationships that could be construed as a potential conflict of interest.

## Publisher's Note

All claims expressed in this article are solely those of the authors and do not necessarily represent those of their affiliated organizations, or those of the publisher, the editors and the reviewers. Any product that may be evaluated in this article, or claim that may be made by its manufacturer, is not guaranteed or endorsed by the publisher.

## Abbreviations

BMI: body mass index
NSAIDs: non-steroidal anti-inflammatory drugs.
